# High Throughput, Absolute Determination of the Content of a Selected Protein at Tissue Levels Using Quantitative Dot Blot Analysis (QDB)

**DOI:** 10.3791/56885

**Published:** 2018-08-21

**Authors:** Xiaoying Qi, Yunyun Zhang, Yuan Zhang, Tianhui Ni, Wenfeng Zhang, Chunhua Yang, Jia Mi, Jiandi Zhang, Geng Tian

**Affiliations:** ^1^Medicine and Pharmacy Research Center, Binzhou Medical University; ^2^Yantai Zestern Biotechnique Co. LTD; ^3^Precision Medicine research center, Binzhou Medical University; ^4^Department of Chemistry - BMC, Uppsala University

**Keywords:** Biochemistry, Issue 138, Quantitative Dot Blot analysis (QDB), immunoblot, high throughput, absolute determination, Elisa Analysis.

## Abstract

Lacking a convenient, quantitative, high throughput immunoblot method for absolute determination of the content of a specific protein at cellular and tissue level significantly hampers the progress in proteomic research. Results derived from currently available immunoblot techniques are also relative, preventing any efforts to combine independent studies with a large-scale analysis of protein samples. In this study, we demonstrate the process of quantitative dot blot analysis (QDB) to achieve absolute quantification in a high throughput format. Using a commercially available protein standard, we are able to determine the absolute content of capping actin protein, gelsolin-like (CAPG) in protein samples prepared from three different mouse tissues (kidney, spleen, and prostate) together with a detailed explanation of the experimental details. We propose the QDB analysis as a convenient, quantitative, high throughput immunoblot method of absolute quantification of individual proteins at the cellular and tissue level. This method will substantially aid biomarker validation and pathway verification in various areas of biological and biomedical research.

**Figure:**
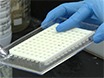


## Introduction

Alongside with the exciting advancements in genomic research in the recent years, biomedical research field also witnesses the significant advancement in proteomic research. With increasing accumulation of biological data at both genomic and proteomic levels, using bioinformatic tools to analyze these data has become the focus of biomedical research in the perceivable future. Consequently, the success of bioinformatical research raises demand for more and better quality of data from the biological and biomedical research community, a task can only be achieved through technique advancement at genomic and proteomic levels.

Mass spectrometry (MS) and immunoblot analysis are two prevailing techniques of protein analysis presently. MS has dominated the proteomic research in the recent years to enable analysis of thousands of individual proteins simultaneously. The immunoblot-based techniques, including western blot and dot blot, on the other hand, have also played a significant role in the protein research even since its invention^1^,^2^,^3^,^4^,^5^. Enzyme linked immunosorbent assay (ELISA)^5^,^6^,^7^ and reverse phase protein microarray (RPPM)^8^,^9^ can be considered the high throughput format of immunoblot analysis. However, all these immunoassay methods, except ELISA, measure the relative expression level of a specific protein. The relative nature of these methods will become a real problem for population studies, as the analysis must be done at the same time, preventing any efforts to increase the pool size through multiple analyses. Furthermore, the results derived from these studies are only semi-quantitative, thus complicating any bioinformatics efforts in the data analysis. Meanwhile, although ELISA is well suited for high throughput absolute analysis of protein samples, this technique seems to meet challenges in complex environments such as cells or tissues because of its low binding capacity and multiplexing^10^.

We have developed an immunoblot method suitable for population studies with the key characteristics of being convenient, high throughput, quantitative, and suitable for absolute determination of protein content, and named this technique quantitative dot blot analysis (QDB)^11^. In this study, we present a detailed protocol for QDB analysis and demonstrate the method by determining the absolute protein content of a specific protein, CAPG, in three different mouse tissues including kidney, spleen, and prostate. We think that this detailed protocol well illustrates the feasibility and convenience of this method, and provide guidance on how to avoid the potential pitfalls in the practice of this method.

## Protocol

All animal procedures were carried out in line with Binzhou Medical University Animal Use Directive and approved by the ethical review board of Binzhou Medical University.

### 1. Sample Preparation

1. Take 50 mg mouse tissue into a 1.5 mL Tube. Add 200 µL lysis buffer (50 mM Hepes, pH 7.4, 137 mM NaCl, 5 mM EDTA, 5 mM EGTA, 1 mM MgCl_2_, 10 mM Na_2_P2O_7_, 1% Triton X-100, 10% glycerol), supplemented with protease and phosphatase inhibitors (100 mM NaF, 0.1 mM phenylmethylsulfonyl fluoride, 5 µg/mL pepstatin, 10 µg/mL leupeptin, 5 µg/mL aprotinin).
2. Homogenize the tissue with a homogenizer for 1 min on ice.
3. Centrifuge for 10 min at 8 000 x g at 4 °C.
4. Collect the supernatant into new tubes (1.5 mL) and take out 1 µL for protein concentration assay with BCA kit. **Note:** All the commonly used lysis buffers for western blot analysis and Dot blot analysis can be used for QDB analysis.

### 2. Determining the Specificity of Antibody

1. Prepare sample lysates (20 µg) in section 1.4, IgG free BSA (20 µg), standard protein (600 pg), for western blot analysis. **Note:** IgG free BSA is used as a negative control and the standard protein is used as a positive control. The specificity of the antibody is demonstrated by showing one band of right size using the western blot analysis.

### 3. Defining the Linear Range of the QDB Analysis

1. Dissolve the standard protein with ddH_2_O. Dilute the protein to a concentration of 1,200 pg/µL.
2. Dilute the concentrated protein from the prepared samples in section 1.4 to 4 µg/µL.
3. Dissolve the BSA with ddH_2_O. Dilute the protein to a concentration of 1,200 pg/µL and 4 µg/µL.
4. A series of dilution of standard protein and prepared samples were prepared with the guidance of **Table 1** and **Table 2**.
5. Mix 10 µL dilution standard protein or dilution prepared samples and 10 µL 2x loading buffer (120mM Tris-HCl pH 6.8, 20% Glycerol, 4% SDS, 0.2% Bromphenol Blue, 200 mM DTT) together.

**Table:** 

	**S1 (0pg/µl)**	**S2 (33.3pg/µl)**	**S3 (100pg/µl)**	**S3 (300pg/µl)**	**S5 (600pg/µl)**	**S6 (1200pg/µl)**
**standard protein(1200pg/µl)**	0	1.39	4.17	12.5	25	50
**IgG free BSA (4pg/µl)**	50	48.61	45.83	37.5	25	0
**total**	50	50	50	50	50	50

**Table 1. Dilution Scheme for standard protein curve.**

**Table:** 

	**X1 (0µg/µl)**	**X2 (0.25µg/µl)**	**X3 (0.5µg/µl)**	**X4 (1µg/µl)**	**X5 (2µg/µl)**	**X6 (4 µg/µl)**
**Sample(4µg/µl)**	0	3.125	6.25	12.5	25	50
**IgG free BSA(4µg/µl)**	50	46.875	43.75	37.5	25	0
**total**	50	50	50	50	50	50

**Table 2. Dilution Scheme for prepared samples**

1. Heat the mixtures at 85 °C for 5 min.

### 4. Process of QDB Analysis

1. **Sample application**
  1. Support the QDB plate to avoid the bottom of the plate touching the surface of the table. For instance, use an empty pipette tip box as support.
  2. Load up to 2 µL of the sample to the center of the membrane at the bottom of the individual unit of the QDB plate.
2. **Drying the plate**
  1. Leave the loaded QDB plate for 1 h at room temperate (RT) or as an alternative leave the loaded plate at 37 °C for 15 min in a well-ventilated space.
3. **Blocking the plate**
  1. Dip the QDB plate in the transfer buffer (0.039 M Glycine, 0.048 M Tris, 0.37% SDS, 20% methyl alcohol) and gently shake the plate for 10 s.
  2. Rinse the QDB plate gently with TBST (Tris-buffered saline, 0.1% Tween 20) three times, and then wash the plate for 5 min in TBST under constant shaking.
  3. Block the QDB plate with blocking buffer (5% nonfat milk in TBST (100 µL/well) for 1 h under constant shaking.
4. **Primary antibody incubation**
  1. Dilute the primary antibody in the blocking buffer at a chosen concentration (from 1: 500 to 1:5 000), and add 100 µL to each individual well of an ordinary 96 well plate. Insert the QDB plate into the 96 well plate and incubate the combined plates either for 2 h at RT or overnight at 4 °C under constant shaking.
  2. Alternatively, place the QDB plate inside a box, and fill the box with blocking buffer 2 to 3 mm above the membrane portion of the plate if the same antibody is used for the whole plate. Add the primary antibody at the chosen concentration, and incubate the plate either for 2 h at RT or overnight at 4 °C by under constant shaking.
  3. Rinse the plate gently with TBST for three times before it is washed with TBST for three times, each time for 5 min under constant shaking.
5. **Secondary Antibody incubation**
  1. Dilute the secondary antibody in the blocking buffer at the chosen concentration (from 1:1, 000 to 1:50, 000), and either aliquot 100 µL/well into a 96 well plate or in a box as described in section 4.4.2, and then incubate the QDB plate inside either the loaded 96 well plate or the box for 1 h at RT under constant shaking.
  2. Rinse the QDB plate gently 3 times with TBST, then wash the plate for 3 times, 5 min each with TBST under constant shaking.
6. **Quantification**
  1. Prepare enhanced chemiluminescence substrate (ECL) by following the manufacturer's instructions.
  2. Aliquot ECL substrate into a 96 well plate (100 µL/well) and insert the QDB plate inside the 96 well plate for 2 min under constant shaking.
  3. Remove the QDB plate from the 96 well plate and shake briefly to remove the excess liquid. Place the plate onto a white microtiter plate.
  4. Turn on the microplate reader, and select "plate with cover" on the user interface before placing the combined plates (QDB plate + white plate adaptor) inside the microplate reader for quantification. **Note:** Make sure to use the white, non-transparent microtiter plate as an adaptor to avoid any interference from the plate. Make sure to choose "plate with cover" to avoid jamming the machine when combined plates (QDB plate+ 96 well plate adaptor) are placed inside the microplate reader.

## Representative Results

The determination of the absolute amount of any protein including the CAPG protein in mouse tissue requires both a specific antibody and a purified protein as standard. The linear range of the analyses of both the protein standard and the lysates also needs to be established prior to any large-scale analysis. The linear range of the antibody is highly dependent on the antibody per se, and the appropriate dilution range of a specific antibody need to be verified by each individual user.

We first determined the specificity of the CAPG antibody in mouse kidney, spleen and prostate tissues using western blot analysis with the negative control (IgG free BSA) and positive control (commercially available CAPG protein) (Figure 1**A**). The anti-CAPG antibody was specific against lysates from mouse spleen, heart, muscle, and prostate (one band detected). Non-specific bands were observed in lysates from kidney and liver. However, in kidney lysate, the non-specific bands were significantly weaker than the specific band, which suggests the antibody is suitable for kidney tissue analysis. Next, the linear range of the protein standard and the amount of lysates for analyses were determined by two side by side dose curves. A commercially available CAPG protein was serially diluted as indicated in **Table 1** based on our past experiences, and the tissue lysates of the mouse kidney, spleen and prostate were also diluted based on the amount of total protein in the lysates determined by a BCA total protein determination kit. Two dose studies were performed side by side and plotted together in Figure 1**B**. The appropriate amount of lysates of mouse kidney, spleen and prostate were chosen based on the QDB signals measured by the microplate reader in arbitrary unit. The original reading for this experiment is shown in **Table 3**.

In the last step, the serially diluted CAPG protein standard and the lysates from mouse kidney, spleen and prostate were loaded onto the QDB plate. In this case, we chose to load 0.3 µg/sample for lysates from mouse kidney and spleen and 1 µg/sample for lysates from mouse prostates analysis, as at these levels, the QDB reading was at least 20-fold over the background while well within the linear range of the analysis based on the dose curve of both the lysates and the protein standard. The plate was subjected to the described QDB protocol before the plate was quantified directly by the microplate reader. Using the serially diluted purified CAPG protein, we could establish a dose-response curve, the equation, and R2 by simple regression analysis using available software (e.g. Microsoft office excel). The QDB signals from lysates of mouse kidneys, spleens and prostates were converted to the absolute amount of CAPG protein in these lysates using the established equation, and the results were corrected by the total protein amount, in this case, 0.3 µg for lysates from mouse spleens and kidneys, and 1 µg for lysates from mouse prostates, for the final concentration of CAPG protein in these tissues as pg/µg. The results are shown in Figure 1**C** with the original reading shown in **Table 4.**

**Figure:**
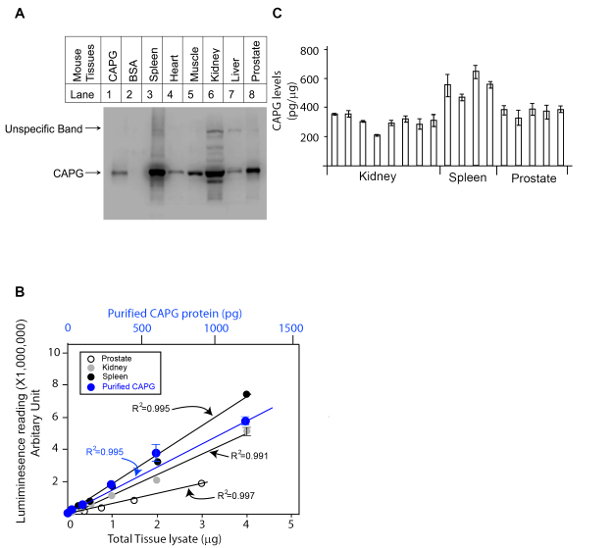

Figure 1**. Representative result of a QDB analysis to determine the absolute CAPG levels in three mouse tissues (kidney, spleen and prostate). A.** Examining the specificity of rabbit anti-CAPG antibody using mouse tissue lysates prepared from Spleen, Heart, Muscle, Kidney, Liver, and Prostate using western blot analysis with the negative control (IgG free BSA) and positive control (commercially available CAPG protein). **B.** defining the linear range of QDB analyses of rabbit anti-CAPG antibody using lysates prepared from prostate, kidney, spleen, and using purified recombinant CAPG protein standard. The results were an average of triplicates. **C.** Absolute determination of CAPG levels in lysates prepared from mouse kidneys, spleens, and prostates. Each bar represents one tissue from an individual mouse. The results were the average of triplicate. Please click here to view a larger version of this figure.


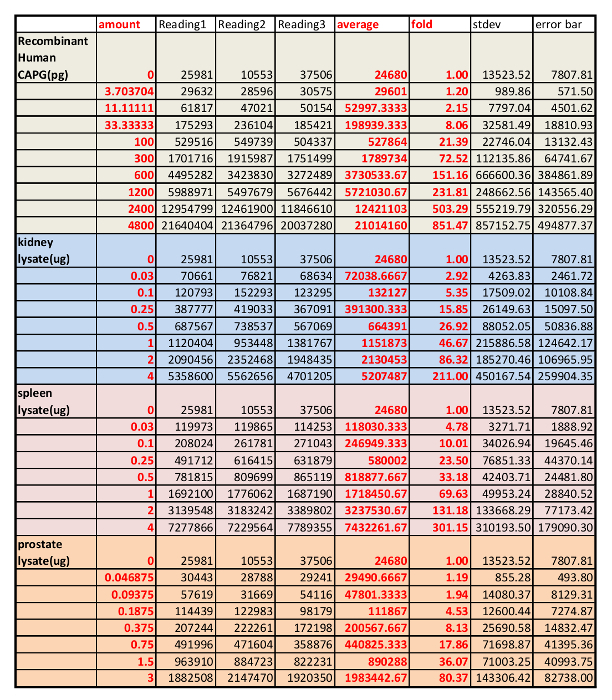
 **Table 3: Result from microplate reader of the dose studies using both serially diluted, pooled lysates from mouse spleens, kidneys, and prostates and purified recombinant CAPG protein.**


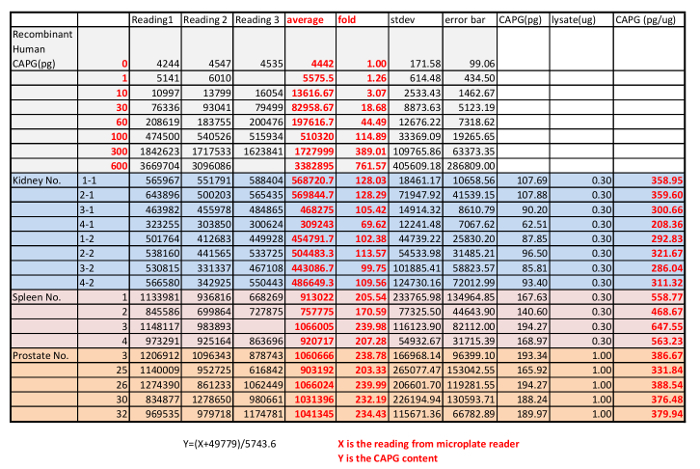


**Table 4: Result from microplate reader of the QDB analysis of the absolute CAPG level in mouse kidneys (8), spleens (4) and prostates (5) using a recombinant CAPG protein as standard.**

## Discussion

Among all the current available immunoassay techniques of protein analysis except ELISA, QDB analysis is the only method to achieve absolute content of a specific protein at cellular and tissue levels in a high throughput format. Although with stable isotope-labeled standard, mass spectrometry is able to achieve absolute quantification of a few proteins, this method is not yet designed for high throughput performance. In this study, we demonstrated the process of QDB analysis to achieve absolute determination of CAPG protein level in mouse tissues including kidney, spleen, and prostate. CAPG levels were found to be between 200 to 360 pg/g in mouse kidney tissues, 460 to 650 pg/g in mouse spleens, and 330 to 390 pg/g in mouse prostate tissues.

Compared to ELISA, QDB analysis requires minimum efforts to be developed in a regular lab. First, the QDB plate is a based on a nitrocellulose membrane to eliminate the coating step in ELISA. Second, QDB analysis only requires one instead of two specific antibodies in a sandwich ELISA. Third, the high binding capacity of nitrocellulose membrane as compared to the ELISA plate surface also allows the membrane to withstand the stringent washing steps typically used in the immunoblot analysis to reduce the background interference. This feature is very useful in analyzing complicated lysates prepared from cells and tissues. In contrast, due to the relatively low binding capacity of ELISA plates, the reduction of the background when analyzing complicated cellular and tissue lysates becomes a real challenge in the developmental process.

QDB analysis can be adopted easily in any lab with the access of a specific antibody. However, it is important to mention that the specificity of the antibody is a relative term, limited by factors including the species, the tissue types and cell types. As shown in Figure 1**A**, CAPG antibody is specific when analyzing lysate from mouse kidney, spleen, and prostate, and yet becomes non-specific when analyzing mouse liver. In fact, we routinely found one antibody to be specific for one tissue type, but not always specific to other tissue type from the same species. Thus, prior western blot analysis is necessary to ensure the specificity of the QDB analysis. In fact, the relative specificity of the antibody may be the cause of false results often associated with ELISA analysis, as no matter how much effort the manufacturing company may have spent to ensure the specificity of the assay, they cannot exhaust all the possible types of samples the users may choose to analyze using their ELISA products.

As with all immunoassays, a potential problem with the QDB analysis method is the lack of consistency of the quality of the commercial antibody. Even if the apparent quality of an antibody from the same company (same catalog number, etc.) is used, there could be large differences from one purchase to another due to batch-to-batch variability. Therefore, unless full confidence is attained in the quality of the antibody available, re-characterizing the antibody upon every purchase is important.

In summary, we provide here a detailed protocol and an example when using the QDB analysis method to achieve absolute quantitative determination of a specific protein at the tissue level. We show that the QDB analysis method is a convenient tool for anyone who is interested in high throughput, quantitative immunoblot analysis. Its ability to achieve absolute determination of the content of a specific protein at a cellular and tissue level also distinguish this technique from traditional immunoblot techniques. This feature allows for the combination and comparison of results from multiple analyses, a step necessary especially in larger population studies, and the realization of relevant association studies at the protein level in the near future.

## Disclosures

The authors Yunyun Zhang, Wenfeng Zhang and Jiandi Zhang are employees of Zestern Biotechniques that produces QDB plates used in this Article. Wenfeng Zhang and Yunyun Zhang declare conflict of interests, and Jiandi Zhang has filed patent applications. The others claim no competing interests.
